# Immune Cell Subtypes and Their Function in the Testis

**DOI:** 10.3389/fimmu.2020.583304

**Published:** 2020-09-30

**Authors:** Sudhanshu Bhushan, María S. Theas, Vanesa A. Guazzone, Patricia Jacobo, Ming Wang, Monika Fijak, Andreas Meinhardt, Livia Lustig

**Affiliations:** ^1^ Department of Anatomy and Cell Biology, Justus-Liebig University Giessen, Giessen, Germany; ^2^ Hessian Center of Reproductive Medicine, Justus-Leibig-University Giessen, Giessen, Germany; ^3^ Departamento de Biología Celular e Histología/Unidad Académica II, Facultad de Medicina, Universidad de Buenos Aires (UBA), Buenos Aires, Argentina; ^4^ Consejo Nacional de Investigaciones Científicas y Técnicas (CONICET), Facultad de Medicina, Instituto de Investigaciones Biomédicas (INBIOMED), Universidad de Buenos Aires (UBA), Buenos Aires, Argentina; ^5^ Medical Research Center, The First Affiliated Hospital of Zhengzhou University, Zhengzhou, China

**Keywords:** testis, immune privilege, macrophages, dendritic cells, T lymphocytes, mast cells

## Abstract

Immunoregulation in the testis is characterized by a balance between immuno-suppression (or immune privilege) and the ability to react to infections and inflammation. In this review, we analyze the phenotypes of the various immune cell subtypes present in the testis, and how their functions change between homeostatic and inflammatory conditions. Starting with testicular macrophages, we explore how this heterogeneous population is shaped by the testicular microenvironment to ensure immune privilege. We then describe how dendritic cells exhibit a tolerogenic status under normal conditions, but proliferate, mature and then stimulate effector T-cell expansion under inflammatory conditions. Finally, we outline the two T-cell populations in the testis: CD4^+^/CD8^+^ αβ T cells and CD4^+^/CD8^+^ Foxp3^+^ regulatory T cells and describe the distribution and function of mast cells. All these cells help modulate innate immunity and regulate the immune response. By improving our understanding of immune cell behavior in the testis under normal and inflammatory conditions, we will be better placed to evaluate testis impairment due to immune mechanisms in affected patients.

## Introduction

**Figure 1:**
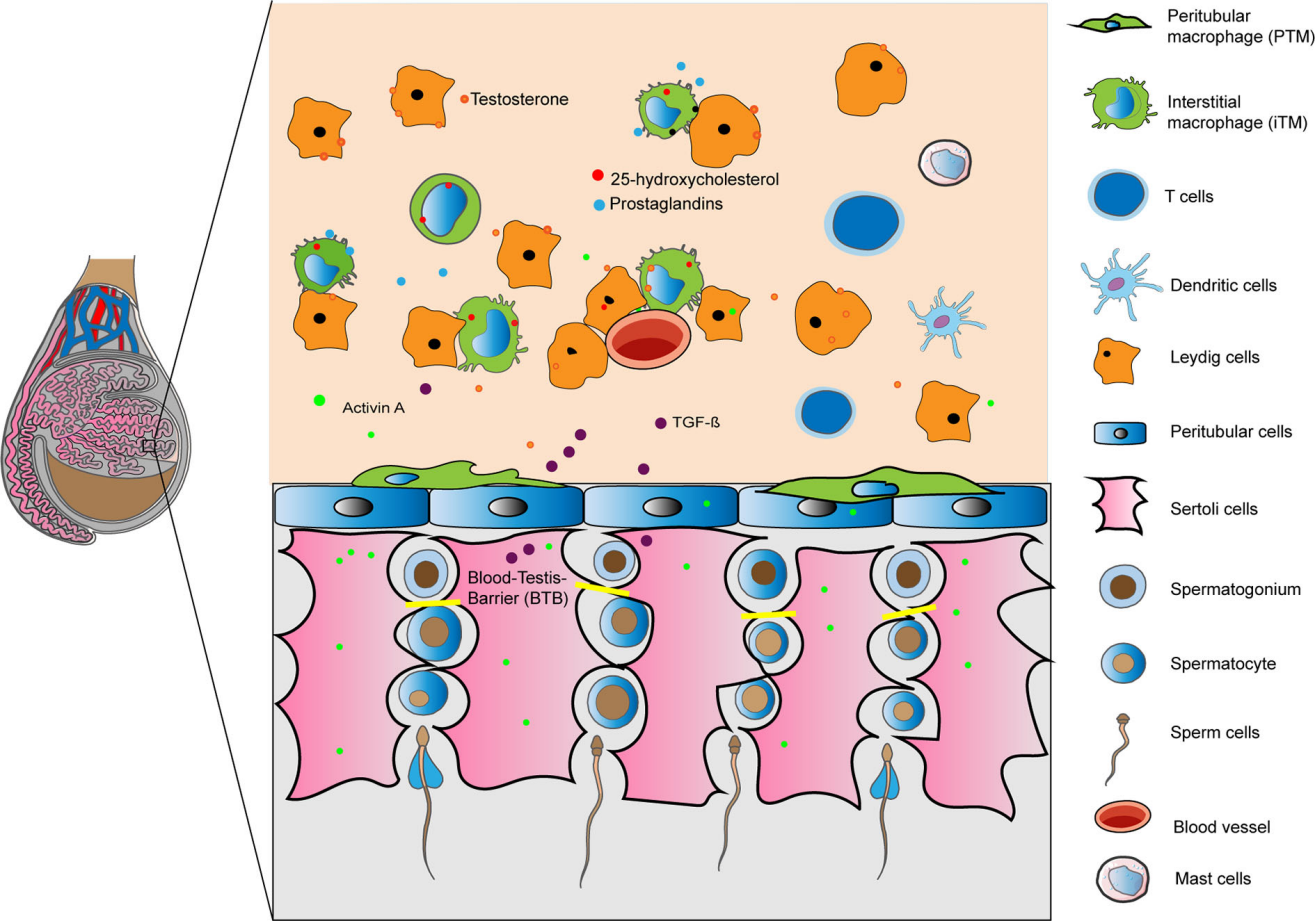
Immunoregulation of the testis is mediated by a combination of structural (blood- testis barrier) and cellular derived factors. Illustration shows in a rodent testis, resident macrophages, tolerogenic dendritic cells, T cells and mast cells in the interstitium interacting with Leydig, Sertoli and peritubular cells releasing factors to create an optimal microenvironment for germ cells. Under inflammatory conditions immune cells are able to respond to invading pathogens.

The mammalian testis is divided into two main compartments: the convoluted seminiferous tubules, where spermatogenesis occurs, and the interstitial space where Leydig cells (LC) produce the male sex hormones (androgens). During spermatogenesis, germ cells divide and mature in close association with somatic Sertoli cells (SC) that extend from the basal membrane to the tubule lumen. Multiple highly specialized cell junctions namely tight, adherens and gap junctions extend between neighboring SC to form a blood-testis-barrier (BTB) that is permissive to the developing germ cells. Bordering the SC and spermatogonia, a basement membrane containing extracellular matrix proteins and a contractile peritubular cells (PTC, also known as myoid cells) sheet divides the seminiferous epithelium from the interstitium. Organization of the PTC sheet varies by species; in rodents, only one PTC layer is seen whereas in humans it typically consists of 5–7 layers (1). Immune cells in the testis are found exclusively in the interstitium, in close association with either PTC or LC, as well as with lymphatic and blood vessels (**Figure 1**). Some authors described that in rodents lymphatic vessels are restricted to the tunica albuginea (2–4), while others reported irregular channels incompletely bound by endothelial cells (5). In contrast, Kitadate et al. (6) described in mice testis parietal lymphatic endothelial cells (LE) that cover the surface of lymphatic space, mainly in the peritubular area close to the PTC layer contributing to the spermatogenic stem cell niche homeostasis through the supply of fibroblast growth factor ligands secreted by LE cells (6). Telocytes, a stromal cell type similarly localized as LE cells, have been recently identified in rodent and human testis (7, 8). It has been suggested a possible role of telocytes in the regulation of lymphatic capillary function (9). The immunomodulatory factor secreted by immune cells, and somatic cells (LC, SC, and PTC), in combination with BTB, forms a unique immunological environment that: (i) protects immunogenic germ-cell-specific neoantigens and transplants from immune attack, and (ii) responds to invading pathogens by eliciting a delicately balanced immune response to protect sensitive germ cells (10, 11).

The functions of the BTB, SC, LC, and PTC in preserving the immune privileged status of the testis has been described in details in previous reviews (11, 12). As such, we rather describe the different immune cells of the testis that maintain the immune privileged status and contribute to the immune response against inflammatory stressors.

## Macrophages

In the broadest sense, macrophages constitute an immune cell population with phagocytic characteristics, ingesting foreign particles and invading pathogens such as bacteria, and clearing cell debris. Given their essential role, macrophages are found in all mammalian organs where they perform immune and nonimmune functions (13). As is the case for other organs, macrophages are by far the most abundant and heterogeneous immune-cell population found in the testis (14, 15). The heterogeneity of these tissue-resident macrophages has been delineated based on cell surface marker expression, anatomical localization, gene expression profiles, ontogeny, and post-natal development. Under normal conditions, most testicular macrophages (TM) express the core macrophage markers F4/80, CD11b, AIF, and CX3CR1 (4, 16). The heterogeneity of the macrophages is evidenced by the differential expression of surface markers CD64 and major histocompatibility complex class II (MHC II), besides their localization relative to other cells within the testis (4, 16).

Under steady conditions, interstitial (iTM) and peritubular (pTM) macrophages predominate (16). iTM are characterized by CD64^hi^MHC^lo^ expression and are found adjacent to LC and blood vessels (**Figure 1**) (4, 16). By contrast, pTM are characterized by CD64^lo^MHC^hi^ expression and are found adjacent to the seminiferous tubules (**Figure 1**) (4, 16). Each TM subpopulation also expresses a unique transcriptional profile, which is indicative of functional specialization. For example, CD64^lo^MHC^hi^ pTM express high levels of antigen presentation genes, such as genes encoding the MHCII — H2Dmb, H2Eb1, and, H2K1. Conversely, CD64^hi^MHC^lo^pTM express high levels of immunosuppressive genes, namely, IL10 and Marco (16).

A recent study used single-cell mRNA sequencing (scRNA-seq) to identify two interstitial macrophage sub-populations of the lung, which were also found in the testis: Lyve1^lo^MHCII^hi^ and Lyve1^hi^MHCII^lo^. These two subpopulations are distinct in localization, phenotype, and gene expression (17). Although TM seem to comprise only two main subsets, in-depth analyses using scRNA-seq and high powered confocal microscopy are needed to characterize the extent of heterogeneity in detail.

Resident macrophages in the brain, liver, and lung that arise embryonically either from the yolk sac or fetal liver; are maintained locally by self-renewal in adult life without notable contribution from blood monocytes (18). In other organs, such as the intestine, dermis, and pancreas, macrophage populations are maintained by replacement from blood monocytes (19–22). However, the exact contribution of embryonic progenitors and blood monocytes that give rise to tissue-resident macrophages in adult life is still controversial. Moreover, the mechanism by which adult tissue resident macrophages maintained in adult life is not yet completely understood.We have made great progress in recent years to unravel the ontogeny and functional heterogeneity of macrophage population in different organs, the origins of TM are still unclear. iTM are thought to originate from embryonic progenitors, whereas pTM seem to arise postnatally from bone marrow derived cells (16); however, the relative contributions of embryonic progenitors to the adult TM and the mechanisms of TM maintenance in adulthood are undefined.

Although all tissue-resident macrophages arise from distinct cellular origin, (23) upon organ entry, tissue-specific signals polarize macrophages to endow them with tissue- specific functions (24). For example, the intranasal transfer of progenitors from either the yolk sac, fetal liver, or bone marrow to the lungs of *Csf2rb^-/-^* mice lacking adult alveolar macrophages resulted in the polarization of macrophage progenitors with functional and transcriptional properties reminiscent of alveolar macrophages. These findings suggest that intrinsic factors rather than cellular origins, might imprint the resident macrophage phenotype (25). The testis microenvironment contains numerous immunomodulatory molecules — namely testosterone, prostaglandins, corticosterone, activin, and 25 hydroxy-cholesterol (25HC) — that might govern TM phenotype and function (**Figure 1**) (14, 26). Indeed, we found that testosterone, prostaglandins, and corticosterone present in the testicular interstitial fluid can polarize GM-CSF-derived macrophages to immunosuppressive macrophages by inducing CD163 and IL10 expression with concomitant low TNF-α production (14). Other molecules, such as 25-HC and activin, are also produced in large amounts in the testis (26, 27). Whether these molecules can influence TM phenotype and function has not been investigated, we speculate that they likely have a role.

Recent data primarily from *in vitro* studies suggest a role for TM in maintaining the immune privileged state of the testis (28, 29). Specifically, TM exhibit anergy to inflammatory stimuli, which serves to protect developing germ cells from the deleterious effects of pro-inflammatory cytokine. The mechanisms underlying this subdued inflammatory response of TM could be based on the low expression of toll like receptor (TLR) signaling genes and impaired ubiquitination and degradation of the NF-ĸB inhibitor IĸBα that ultimately inactivates the inflammatory NF-ĸB signaling pathway (28).

TM are immunoregulatory, secreting high levels of IL10, and producing low levels of TNF-α and nitric oxide (NO) upon stimulation with the TLR4 ligand, lipopolysaccharide (LPS) (28, 29). In addition, TM suppress T-cell proliferation and activation, and induce naive T-cell differentiation into immunosuppressive regulatory T cells (Tregs) (14, 29). The TM immunoregulatory phenotype seems to be essential to preserve normal testis homeostasis: high TNF-α production impairs LC function and can breakdown the BTB, exposing the germs cell to cytotoxic inflammatory cytokines that ultimately impairs spermatogenesis (30). High NO production can also negatively affect LC function and consequently, steroidogenesis (31).

Besides acting as immune sentinel cells, TM are also equipped to perform testis-specific functions to maintain normal homeostasis, including: (1) supporting steroidogenesis by producing 25HC, (2) promoting spermatogenesis by expressing sp?A3B2 ?>ermatogonial proliferation- and differentiation-inducing factors such as colony stimulating factor 1 (CSF1) and enzymes involved in retinoic acid (RA) biosynthesis, and (3) guiding testis embryonic development by aiding blood vessel and spermatic cord formation (4, 26).

The number of TM increases during testis inflammation, as observed following acute LPS stimulation or in experimental models of murine chronic autoimmune epididymo-orchitis (EAO) induced by immunization with testis homogenate and adjuvants or pathogen-induced (*E. coli*) inflammation (epididymo-orchitis) (32–35). During LPS-induced inflammation, a transient influx of monocytes into the testis occurs that resolves after 72 h; whereas no changes in the number of TM was observed (32). Similarly, following *E. coli*-induced epididymo-orchitis, inflammation in the testis quickly resolves after the initial infiltration of immune cells and impairment of spermatogenesis. However, without therapeutic intervention, inflammation of the epididymis continues to remain (35). These observations suggest that the testis has a remarkable ability to resolve inflammation *via* mechanisms likely involving TM. Now, further studies are required to investigate how TM resolve inflammation and promote tissue repair.

We have gained mechanistic detail underlying the role of TM in resolving inflammation from the experimental autoimmune orchitis (EAO) model. Here, the number of TM subsets CD68^+^CD163^-^ and (CD68^+^CD163^+^) increases progressively from the end of the immunization period to the severe orchitis stage, with minor changes in the number of resident CD163^+^ TM (33). At the same time, TM release large amounts of the pro-inflammatory mediators TNFα, IL6, IFNγ, and NO but not IL10 and GM-CSF (33, 36, 37). Infiltrating macrophages, but not resident CD163^+^ TM, express IL6, upregulate MHCII and reduce TNFα expression (33). Neither infiltrating (CD68^+^CD163^-^), intermediate (CD68^+^CD163^+^) nor resident (CD68^-^CD163^+^) TM up-regulate iNOS expression during EAO (37). The increase in NO production by TM during EAO mainly results from the large percentage of infiltrating and intermediate TM expressing iNOS; resident TM contribute to NO production to a lesser extent. These events, as well as changes in other immune and nonimmune cell functions, ultimately disrupt testicular immune privilege and impair spermatogenesis and steroidogenesis (38).

The consequences of perturbed TM function are evident in infertile patients with hypospermatogenesis and Sertoli cell-only syndromes. Namely, patients present with an increased level of the TM-derived pro-inflammatory cytokines TNFα, IL1α, and IL1β (39, 40). Interstitial infiltration of activated macrophages and TNFα production is also a common feature in other models of testicular damage, such as testicular torsion (41), and in two models of transgenic male mice, one ectopically expressing humanP450 aromatase (AROM+), and the Tyro3, Axl and Mer (TAM) receptor tyrosine kinase triple knockout (42).

## Dendritic Cells

Dendritic cells (DC) are “professional” antigen-presenting cells and a cellular component of the adaptive immune system. DC constitute a heterogeneous population that includes classical DC, plasmacytoid DC, and monocyte-derived DC originating from hematopoietic stem cells in the bone marrow. DC are found in lymphoid and nonlymphoid tissues and have a role in T-cell activation and tolerance induction, both of which depend on many environmental signals (43). We found that normal rat testis and testicular draining lymph nodes (TLN) contain CD103^+^ DC that express MHCII and B7 costimulatory molecules. However, functional data from *in vitro* assays revealed that these DC are unable to stimulate naïve T-cell proliferation (36, 44, 45). This finding suggests that DC support the immune privilege status of the testis as they adopt a tolerant status in the physiological testicular microenvironment.

DC isolated from normal testis also express the chemokine receptor CCR2, which is a marker of immature resident DC. Gao et al. showed that Sertoli cells may promote the differentiation of these immature DC into tolerogenic DC since mice prepuberal Sertoli cell-conditioned of mice DC down-regulate the expression levels of costimulatory molecules and decrease T-cell priming (46).

During EAO, CD103^+^ MHCII^+^ CD80^+^ CD86^+^ DC isolated from the testis and TLNs can activate T cells and produce IL12p70 (44, 45). Bioactive IL12p70 can bias activated T cells in favor of an inflammatory Th1 response (47). Moreover, DC isolated from EAO testes show an upregulation of chemokine receptor CCR7 expression, which directs DC migration to the TLN (45). In fact, mature CD103^+^ DC accumulate in the lymph nodes (LN) that drain the EAO testis (44).

Based on our knowledge in the rat model thus far, we propose a putative model by which CD103^+^ DC promote the induction and progression of orchitis. In the LN draining the immunization site, DC present orchitogenic antigens on their surface in the context of MHCII, then prime naïve T cells and polarize them toward effector functions. DC-sensitized T cells migrate to the testis, where they are attracted by local chemokines (CCL2 and CCL3) secreted by antigen presenting cells and somatic cells and contribute to testicular inflammation causing tissue damage (48, 49). Testicular DC take up spermatic antigens from the impaired seminiferous tubules to undergo immunogenic maturation, and subsequently travel to the TLN *via* the lymphatic system; alternatively, they might prime naïve T cells *in situ*. Consequently, this process would increase manifold in response to inflammatory signals leading to chronic orchitis.

## Lymphocytes

Depending on environmental signals, T cells commit to effector or regulatory lineages with opposing functions leading to inflammation or dominant immunologic tolerance (50). Flow cytometric analysis of the leukocytes present in the normal rat testis showed that nearly 25% of these cells are CD3^+^ T cells, and that the percentage of CD8^+^ T cells is 4-fold greater than that of CD4^+^ T cells. Most CD25^+^ T cells are found within the CD8^+^ subset (51). Both subsets express proinflammatory mediators, such as TNFα, IFNγ, and FasL. A similar percentage of cells within the CD4^+^ and CD8^+^ T cell subsets express IFNγ; however, CD8^+^ T cells are the main producers of FasL and TNFα. Testicular T cells expressing IL4 are occasionally observed under normal conditions (52, 53).

The presence of Foxp3^+^ Tregs in the normal testis is well established (54–56). In the rat testis, ~2% of the cells within the CD4^+^ and CD8^+^ T-cell subsets express CD25 and Foxp3 (51). This percentage rises to 4% in the TLN (57). Most of these cells show a memory phenotype and produce TFG-β. Functional analysis of CD4^+^CD25^+^Foxp3^+^ Tregs isolated from the TLN showed that these cells produce a potent proliferative response toward spermatic antigens and exert suppressive effects that prevent conventional T-cell proliferation (57). These results support that Tregs are activated *in vivo* by antigens from the seminiferous tubules. In fact, Tung et al. demonstrated that non sequestered germ cell antigens egressed from seminiferous tubules, enter the interstitium, and induce Tregs challenging the long-standing dogma that all germ cell neo-antigens are sequestered from the immune system (58). These phenomena elicit the expansion of Tregs in the TLN, where they may exert a basal and permanent suppression of auto-reactive T cells, thereby maintaining the tolerogenic environment (57, 58).

Under inflammatory conditions, pathogenic T cells can overwhelm the suppressive mechanisms of Tregs by altering the balance in favor of an autoimmune response (51). For example, during EAO, CD4^+^, and CD8^+^ T cells producing TNF-α, IFNγ, and FasL infiltrate the testis. Th1 and Th17 subsets serve as co-effector cells that govern the early stages of the disease whereas CD8^+^ T cells producing Th1 and Th17 cytokines are relevant to establish chronic inflammation (51–53). Although Tregs accumulate in the testis and in the TLN, and despite that Tregs from the TLN are more effective at suppressing T-cell proliferation than their normal counterparts, these Tregs are unable to prevent germ cell attack. One proposal for this paradigm is that cytokines in the inflammatory milieu might inhibit Tregs *in vivo*, compromising their function at inflammation sites (56, 57).

## Mast Cells

Mast cells (MC) are tissue resident immune cells with heterogeneous phenotypes localized within the subalbuginea area near blood vessels in rodents and/or in the interstitial compartment in humans (**Figure 1**). MC derive from CD34^+^ hematopoietic progenitor cells, differentiating initially in the bone marrow and then locally in the specific organ that they have migrated to under the influence of estrogens (59). MC have diverse roles in innate immunity, tissue homeostasis and remodeling, and adaptive immunity. Pre-synthesized substances such as histamine, chymase, tryptase, carboxipeptidase A, and TNFα are stored in granules and are released immediately after MC activation (60). TNFα, IL6, and IL1β are synthesized *de novo* after MC activation (61). Direct interactions with autoreactive T cells may activate MC, inducing degranulation, and cytokine production.

During inflammation (such as in patients with defective spermatogenesis, varicocele, infertility, or EAO and testis torsion models) the number of MC increases. Here, the serine protease tryptase enhances fibroblast proliferation and collagen synthesis, inducing fibrosis of the seminiferous tubules (62). MC might also regulate fibrosis by activating matrix metalloproteinases (MMP) and tissue MMP inhibitors (63).

In the EAO model, MC mainly accumulate around the ST and especially the surrounding granulomas (64). Tryptase released by these MC activates proteinase-activated receptor-2 (PAR2) that is expressed by peritubular cells and TM to induce cell proliferation and cytokine production (64). Moreover, PAR2-derived peritubular cells drive an increase in the expression of inflammatory mediators MCP1, TGFβ2, and cyclooxygenase COX2.

Analyses of human testis biopsies from infertile patients found an increase in the number of MC within the walls of the seminiferous tubules; many of these cells were active and expressing tryptase (65). These MC were often localized near to spermatogonia and Sertoli cells, suggesting that MC might affect spermatogonia that express PAR2, possibly *via* their secreted products (65).

## Conclusions and Future Perspectives

Immune cells have essential roles in maintaining testicular homeostasis by dampening the inflammatory response and supporting normal physiological functions. The attenuated inflammatory response of testicular immune cells, particularly TM, is essential as the testis is an immune privileged organ. Any inflammatory response can severely damage testicular function — namely steroidogenesis and spermatogenesis. Although substantial progress has been made in understanding testicular immune cell function, more detailed investigations are now required to delineate the interactions between these immune cells and neighboring nonimmune cells. The mechanisms underlying how immune cells help to resolve inflammation and promote tissue repair are to be studied in depth. Advances in this area will improve our understanding of male infertility problems and will pave the way for the development of innovative therapeutics.

## Author Contributions

All authors contributed to the article and approved the submitted version.

## Funding

The support of the Deutsche Forschungsgemeinschaft (DFG) for the International Research Training Group (GRK 1871/2-1) on ‘Molecular pathogenesis of male reproductive disorders’ and the Faculty of Medicine of the Justus-Liebig-University of Giessen is gratefully acknowledged. SB is supported by DFG (BH93/1-4). The support from the University of Buenos Aires and CONICET is greatly acknowledged.

## Conflict of Interest

The authors declare that the research was conducted in the absence of any commercial or financial relationships that could be construed as a potential conflict of interest.
